# Social Environment and Control Status of Companion Animal-Borne Zoonoses in Japan

**DOI:** 10.3390/ani2010038

**Published:** 2012-02-15

**Authors:** Hiromi Takahashi-Omoe, Katsuhiko Omoe

**Affiliations:** 1 Science and Technology Foresight Center, National Institute of Science and Technology Policy, Kasumigaseki 3-2-2, Chiyoda-ku, Tokyo 100-0013, Japan; 2 Disease Control and Environmental Sciences, Department of Veterinary Medicine, Iwate University, Ueda 3-18-8, Morioka, Iwate 020-8550, Japan; E-Mail: omo@iwate-u.ac.jp

**Keywords:** companion animal-borne zoonoses, Infectious Diseases Control Law, Rabies Prevention Law, emerging zoonoses

## Abstract

**Simple Summary:**

The risk of companion animal-borne zoonoses has been rising in Japan with the tendency for increasing number of households to ever-growing numbers and varieties of animals as pets. In response, the Japanese government has implemented measures for the domestic and border control of zoonoses. However, it is impossible to determine whether these measures have adequately controlled the transmission of companion animal-borne zoonoses, due to a lack of (i) direct evidence linking companion animal involvement in disease and (ii) understanding of current trends in disease outbreak. Active surveillance should be conducted on a national level to collect the data necessary to make this determination and identify these trends.

**Abstract:**

Changing social and environmental factors have been the cause of an increase in the number and variety of animals are being imported into Japan. Moreover, the number of Japanese households are keeping companion animals has also risen. These factors, along with the high density of the Japanese population and the low percentage of registered dogs, have increased the risk of animal-to-human transmission of zoonoses. To control zoonosis outbreaks, the Japanese government has implemented a three-stage approach for the border control of zoonoses and has stipulated the monitoring and reporting of eight companion animal-borne zoonoses under the Rabies Prevention Law and the Infectious Diseases Control Law. The fact that no case of human and animal rabies has been reported over the past 50 years indicates that these measures are highly effective in preventing rabies transmission. Although it is known that the total number of possible companion animal-borne zoonosis outbreaks decreased between 2005 and 2009 when compared with numbers between 2001 and 2004, the number of zoonosis cases that can be attributed to transmission by companion animals remains unclear. Active surveillance should be conducted on a national level to collect the data necessary to determine this number and identify trends in companion-animal transmitted diseases. Using the data collected, regulation systems should be evaluated to determine whether they have met reasonable goals and policy planning conducted for the control of emerging diseases.

## 1. Introduction

Companion animals play an important role in the lives of many people in many countries, including Japan. Indeed, Japan has recently experienced a boom in pet ownership, with a variety of species, from dogs and cats to birds and reptiles, being sold from pet shops to Japanese families, with whom they live as veritable members. However, given that every form of animal-human contact carries an inherent risk of pathogen transmission [1], this trend raises the concern that increasingly close contact with companion animals may lead to increased risk of zoonosis outbreaks in the country.

Due to its successful systems of rabies prevention and control, Japan has remained one of the few rabies-free countries. Drawing on lessons learned in the past, central and local governments, veterinarians, health professionals, and researchers in Japan have endeavored to prevent and control the outbreak of common companion animal-borne zoonoses, such as rabies and psittacosis, under the Rabies Prevention Law (first enacted as Law No. 247 in August 1950; last amended as Law No. 160 in December 1999) [2] and the Law Concerning the Prevention of Infectious Diseases and Medical Care for Patients with Infections, commonly known as the Infectious Diseases Control Law (first enacted as Law No. 114 in October 1998; last amended as Law No. 73 in June 2008) [3]. The former law, which targets dogs, cats, and other animals susceptible to rabies that have a high potential to infect humans, aims to improve public health by preventing the outbreak of rabies and controlling its spread in the event of an outbreak. The latter law complements the aim of the former by preventing and controlling the spread of infectious diseases, including zoonoses, among humans by requiring that humans and animals found to be infected with certain diseases be kept under surveillance.

This study aimed to determine the current status of companion animal-borne zoonosis prevention and control in Japan by investigating the social environment in which companion animals live in Japanese society, the current regulatory framework for the prevention and control of zoonosis outbreaks and the extent to which this framework has been successful. As there is no standard definition of *companion animal*, with some agricultural species, such as horses, considered companion animals by some groups, the analysis was limited to common household pets, specifically dogs, cats, rodents, birds, and reptiles.

## 2. Status of Human-Animal Contact in Japan

As described below, the Japanese population maintains contact with rising numbers and varieties of companion animals, a result of recent lifestyle changes and social trends. Such increased contact, particularly given the relatively high population density in Japan, leads to an increased risk of acquiring zoonotic infection from companion animals. The high incidence of dog bite injuries and low percentage of dog registration and vaccination in the country are additional indicators of increased risk of zoonotic infection. In recognition of this risk, the Japanese government has endeavored to increase knowledge of hygienic management and awareness of companion animal-borne zoonoses among the population to prevent disease outbreak and spread in Japan.

### 2.1. Human Contact with an Increasing Number of Animals

Over the past several decades, the demand for companion animals as family members has increased among the Japanese population, leading to closer contact with a greater number and variety of companion animals, and a concomitant increased risk of infection with animal-derived zoonoses. The desire for a greater number of companion animals can be understood against the backdrop of the current state of Japanese society, in which the number of children per household has decreased while the number of households with only one or two members, particularly among the elderly, has gradually increased due to the falling birthrate and the rapid aging of the population [4]. In fact, the increase in the number of companion animals has become recognized to such an extent that it has become a research concern of the Ministry of Health, Labour and Welfare and the Ministry of Environment. According to a survey conducted by the Ministry of Health, Labor and Welfare to collect data for formulating the *2006 Guidelines On All-Out Hygienic Control of Pets* (FY 2003–2005), approximately 10 million dogs were living as companion animals in Japanese households in 2000, and this number has subsequently increased [5]. Analysis of annual data regarding domestic dogs and cats gathered by the Ministry of Environment indicated a 1.2-fold increase in the number of dogs (10,054,000 to 12,322,000) and a 1.5-fold increase in the number of domestic cats (6,538,000 to 10,021,000) living in Japanese households between 2000 and 2009 [6], as well as a 1.2-fold increase in the number of household dogs registered under the Rabies Prevention Law (5,645,000 to 6,880,000) [7].

### 2.2. Human Contact with an Increasing Variety of Species

The greater variety of species kept as companion animals has led to concerns regarding the increased risk of zoonosis outbreak. These concerns are particularly salient regarding reptiles in light of recent reports of transmission of salmonellosis infection to children from imported red-eared slider turtles and iguanas [8,9]. The Japanese Ministry of Finance reported that almost all of the approximately 400,000 turtles imported annually between 2006 and 2009 [10] originated from the United States, where 74,000 reptile- or amphibian-associated salmonellosis cases are estimated per year [11]. Among all imported reptiles, the red-eared slider turtle is very popular, with about 200,000 having been imported annually since 2005 for sale under the name of *green turtles* [12]. According to a 2005 Internet survey conducted by the Ministry of Environment, about 20% of the 1,099 respondents reported having experience with keeping a red-eared slider turtle as a pet [13]. Together, these findings indicate that the risk of *Salmonella spp.* infection from imported reptiles is considerably high [9], as can be inferred from the data regarding outbreaks of human salmonellosis presented in Section 4.2.

### 2.3. High Density of the Japanese Population

The density of the Japanese population is relatively high, having been found in 2010 to be 343 individuals/km^2^, the seventh most densely populated country in the world [14]. This factor, in addition to that of increased importation of companion species, increases the risk of accelerated transmission of zoonoses throughout the population [4].

### 2.4. Low Percentage of Registered Dogs

The Japanese government is concerned with the relatively low percentage of dogs registered and vaccinated against rabies [15]. According to data reported in 2009, 5,112,401(74%) of 6,880,844 registered domestic dogs were vaccinated [16]. However, since the percentage of registered dogs is assumed to be approximately 58% of the total number of dogs in Japan, as described in *2.1* (6,880,000 registered out of total 12,322,000), the percentage of vaccinated dogs may actually be less than 40%. As describe above, Japan has remained one of the few rabies-free countries. However, given that the World Health Organization (WHO) recommends immunization coverage of at least 70% to control canine rabies in areas where the disease is endemic [17] and illegal importation of rabid animals remains a possibility, Japan faces an undeniable risk of domestic rabies outbreaks [15].

### 2.5. Dog Bite Accidents

National dog bite data in Japan are published under provincial regulations in accordance with the Rabies Prevention Law. These regulations stipulate that a dog bite injury be reported to a local healthcare center after the incidence and that an examination of whether the dog is rabid by a veterinarian be made. According to national data collected at local healthcare centers, approximately 5,000 dog bite injuries were reported annually between 2005 and 2009: 5,275 in 2005, 5,315 in 2006, 5,500 in 2007, 4,950 in 2008, and 4,940 in 2009 [18]. Such a large number of injuries, together with the low percentage of dogs registered and vaccinated against rabies, as described in Section 2.4, increases the risk of domestic rabies outbreaks, as well as of pasturella infection and capnocytophagosis (discussed in Section 4.3) by dog bite, cases of which have been reported in the past.

## 3. Regulatory Framework for Control of Companion Animal-Borne Diseases in Japan

In light of the increased risk of the outbreak of companion animal-borne zoonoses, as described above, the Japanese government has endeavored to control the diseases in both human and animal populations. Two means by which it has done so involves conducting a 3-stage restriction of certain imported species for zoonosis control at the border and stipulating the immediate reporting of eight companion animal-borne zoonoses after diagnosis, as per the Rabies Prevention Law and the Infectious Diseases Control Law. The following sections describe these measures in greater detail.

### 3.1. Regulatory Framework for Border Zoonosis Control

**Figure 1:**
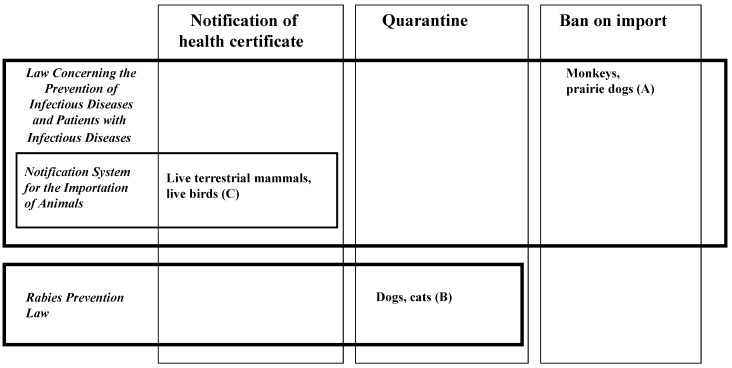
Regulatory framework for the restriction of imports of companion animals into Japan. This figure focuses on possible common household pets as dogs, cats, rodents, and so on. Other than these animals, these laws also cover wild animals and animals used for research or exhibition. Refer to [4] to understand the whole picture of the border zoonosis control in Japan.

To prevent the introduction of zoonoses by animals imported into Japan, the government has restricted animal import by banning the import of certain species, requiring the quarantine of certain other species, and/or requiring the submission of health certificates. Figure 1 shows the regulatory framework for the restrictions on animals that can be imported as companion animals [4].

The import of monkeys and prairie dogs, which had once been imported as companion animals, has been banned under the Infectious Diseases Control Law since 2005 to prevent transmission of plague by prairie dogs and Ebola hemorrhagic fever and Marburg disease by monkeys (Figure 1(A)). As a result, there have been no reports of animals infected with these diseases in Japan since 2005. Under the Rabies Prevention Law (Figure 1(B)), imported dogs and cats must be quarantined for a period ranging from 12 hours to 180 days, depending on the status of rabies outbreaks in the animal’s region of origin and the time necessary for preparation of the required certification. Detention for 12 hours is applicable to dogs and cats imported directly from rabies-free regions (designated regions) and dogs and cats vaccinated and inspected in regions other than designated regions [15]. The designated regions are Taiwan, Iceland, Sweden, Norway, areas of the United Kingdom (Great Britain and Northern Ireland), Australia, New Zealand, Fiji Islands, Hawaii, and Guam (designated on 7 June 2005). Under the quarantine system, which we described in detail in our previous report [15], the number of dogs and cats imported into Japan decreased between 2003 and 2009, as shown in Table 1 [19].

**Table 1:** Annual number of imported and quarantined dogs and cats. The numerical data show the number of animals.

Animal species	2003	2004	2005	2006	2007	2008	2009
**Dogs**	16,892	14,376	8,309	8,099	7,281	6,591	6,391
**Cats**	2,457	2,611	1,635	1,655	1,601	1,591	1,700

Except for that of the species managed by the above regulations, the import of terrestrial animals, including rodents and birds, has been documented since 2006 under the Notification System for the Importation of Animals, which has been enforced since September 2005, as shown in Figure 1(C). This system requires the submission of health certificates for imported animals that had been issued by the government authorities of the exporting country to a quarantine station of the Ministry of Health, Labor and Welfare. The certificates must declare the animals to be free of certain diseases, such as rabies in all species of mammals, plague and tularemia in rodents, and West Nile virus infection and the highly pathogenic avian influenza in birds. Table 2 shows the number of animals of the Rodentia species, specifically mice and hamsters, and of the Psittaciformes species, specifically parrots and parakeets, for which health certificates were submitted from 2006 to 2010 [20]. As can be observed, the number of animals of the Psittaciformes species that were imported decreased remarkably between 2006 and 2010 while that of the Rodentia species remained stable.

**Table 2:** Annual number of animals imported with health certificates. The numerical data show the number of animals.

Animal species	2006	2007	2008	2009	2010
*Rodentia*	456,139	424,431	423,556	416,638	426,946
*Psittaciformes*	28,871	24,562	28,339	17,678	4,178

### 3.2. Regulatory Framework for Rabies Control in Humans and Companion Animals

Except for three cases of imported human rabies in 1970 and 2006, Japan has been free of rabies for approximately 50 years. As shown in Table 3, the last cases of human and animal rabies in Japan were reported in 1954 and 1957 [21].

The elimination of human and animal rabies in Japan can be attributed to the implementation of four countermeasures: (1) daily administrative systems for domestic dogs under the Rabies Prevention Law, (2) border control as a form of import quarantine under the Rabies Prevention Law, (3) the 2*001 Guidelines on Rabies Countermeasures*, and (4) awareness campaigns conducted by related organizations. As the border control of rabies was discussed in section 3.1, the other three countermeasures are discussed in the remainder of this section.

Daily administrative systems for domestic dogs were particularly strengthened under the Rabies Prevention Law [22]. All dog owners are required to register their dogs with the head of the nearest local government once in the lifetime of the animal, ensure that their dogs wear a license tag after registration, and vaccinate their dogs against rabies once a year. After vaccination, the owner must take the vaccination certificate from the veterinarian who administered the vaccine to the head of the nearest local government, and then attach a certification tag to their dogs. Local governments are responsible for managing the registration and vaccination of dogs and assigning veterinarians responsible for identifying and detaining unregistered or unvaccinated dogs when found. Since April 2007, improved standards regarding the licensing and certification of vaccinated dogs have been enforced, including the miniaturization of license and vaccination certification tags so that they can be attached to smaller dogs and allowing local governments to choose the shape of the license and certification tags.

**Table 3:** Annual number of animal and human rabies outbreaks in Japan. Numbers in parentheses refer to cases of feline rabies.

Year	Number of canine rabies cases	Number of feline rabies cases	Number of human rabies cases	Remarks
1949	614	10	76	
1950	867	29	54	Beginning of enforcement of the Rabies Prevention Law
1951	319	3	12	
1952	232	0	4	
1953	176	0	3	
1954	98	0	1	
1955	23	0	0	
1956	6	0	0	
1957	0	1	0	
~				
1970	0	0	1	Imported human rabies case
~				
2006	0	0	2	Imported human rabies cases
2007	0	0	0	Enforcement of improved standards regarding the licensing and certification of vaccinated dogs
2008–2011	0	0	0	

As the standard for preventing and controlling rabies, the *2001 Guidelines on Rabies Countermeasures* has greatly contributed to rabies control [23]. Consisting of a comprehensive handbook for addressing an outbreak or suspected outbreak of rabies in Japan, the guidelines establish measures to guide governmental, medical, and other responsible institutions to take suitable initial actions. According to the guidelines, the two human rabies cases identified in 2006 were stringently controlled in terms of the initial response taken to prevent a rabies outbreak and the medical treatment of the cases, although both individuals died of the disease [15].

Regardless of the above regulations, the low percentage of registered (approximately 58%) and vaccinated (less than 40% of) dogs, as described in Section 2.4, is cause for concern. These low percentages might be due to the nature of the current regulatory systems, which do not cover stray and feral dogs in Japan, or to the attitude of dog owners, who consider rabies to have been eradicated in Japan, and therefore do not believe that their animals need to be vaccinated. In response, the Japan Veterinary Medical Association (JVMA) [24], the Society for the Clinical Study of Rabies, and the Science Council of Japan have conducted campaigns to encourage dog owners to keep their animals immunized against rabies and promote public understanding and cooperation regarding rabies vaccination.

### 3.3. Regulatory Framework for Companion Animal-Borne Disease Control in Humans

The law responsible for preventing and controlling the transmission of infectious diseases from companion animals to humans is the Infectious Disease Control Law. This Law establishes five categories of disease. Infectious Disease Categories 1–4 consist of those needing to be reported promptly after diagnosis by a physician (Category 1 includes the highest-risk diseases) and Infectious Disease Category 5 consists of diseases that should be surveyed nationally.

Of the 52 zoonoses designated for the monitoring of outbreaks [4], eight can be companion animal-borne diseases, with seven—brucellosis, leptospirosis, psittacosis, Q fever, rabies, cryptosporidiosis, and giardiasis—having been reported in humans and one—echinococcosis—in humans and dogs. These possible companion animal-borne zoonoses are classified as “Category 4” and “Category 5.” Physicians suspecting that any patient has been infected with one of these eight diseases must immediately report the patient information to a nearby healthcare center, where it will be reported to a prefectural governor. Such reporting allows the National Infectious Disease Surveillance Center to assemble and analyze the data and announce the results thereof to health professionals. As data regarding the eight companion animal-borne zoonoses are reported by physicians based on Ministry of Health, Labor and Welfare notification standards, and are based on description of clinical characteristics and results of laboratory tests, the reported number of outbreaks is highly reliable. For example, psittacosis is diagnosed by evaluation of both clinical symptoms, such as high fever accompanied by chills, and respiratory symptoms, as well as isolation of *Chlamydophila psittaci* or detection of its gene or antibody.

In view of the number of outbreaks of the eight companion animal-borne zoonoses in humans between 2001 and 2009, giardiasis was the most frequently reported zoonosis during this period, followed by echinococcosis and psittacosis (Table 4) [25]. Except for that of these three zoonoses, the number of outbreaks of each zoonosis did not change markedly in humans over this period. Although the total number of companion animal-borne zoonosis outbreaks in humans showed a decreased trend between 2005 and 2009 when compared with numbers between 2001 and 2004 (mean ± standard deviation 166.8 ± 10.6 *vs.* 259.1 ± 53.9, respectively), it did not change by a significant level. Meanwhile, a small number of echinococcosis outbreaks in dogs was reported between 2005 and 2009. Although outbreaks of the eight zoonoses may not always be associated with companion animal ownership, the lack of active surveillance of zoonosis transmission at the national level prevents determination of the true number of outbreaks attributable to companion animal-borne zoonoses.

In order to examine the role of companion animals in the eight diseases, the prevalence of leptospirosis and echinococcosis in humans and dogs was compared, diseases for which both human and animal data are officially published. Despite analysis of the data—53 human cases (Table 4) *vs.* 358 dog cases [26] and 141 human cases *vs.* 11 dog cases (Table 4) of leptospirosis and echinococcosis, respectively, between 2004 and 2009—it cannot be determined whether companion animals were directly involved in disease transmission.

**Table 4:** Annual number of zoonosis outbreaks in humans and companion animals. Note: ND = not surveyed, since the disease was not required to be reported at that time. * Human shigellosis from “Category 3” is not included because the disease is mostly transferred from contaminated food and water, exceptionally rarely via monkeys. Also, shigellosis in monkeys designated as the “companion animal-borne zoonosis reported by veterinarians” is not included because monkeys have not been imported as companion animal (see Section 3.1) and the animal is not considered as a common household pet in Japan. The latest (2008) amended Infectious Diseases Control Law established the new categories of pandemic human influenza and relevant infections, designated infectious disease (a disease that can be monitored as needed by government ordinance), new infectious disease, and target disease for syndrome-based surveillance (a disease with fever and respiratory symptoms or that results in fever and rash or vesicle for reasons unknown) [3].

	2001	2002	2003	2004	2005	2006	2007	2008	2009
**Companion animal-borne zoonoses reported by physicians**									
**Category 1 **	No companion animal-borne zoonoses belong to this category								
**Category 2**	No companion animal-borne zoonoses belong to this category								
**Category 3**	No companion animal-borne zoonoses belong to this category *								
**Category 4**									
Brucellosis	0	1	0	0	2	5	1	4	2
Echinococcosis	15	10	21	26	20	20	25	23	27
Leptospirosis	ND	ND	1	18	17	24	35	43	16
Psittacosis	35	54	44	40	34	22	29	9	21
Q fever	42	47	9	7	8	2	7	3	2
Rabies	0	0	0	0	0	2	0	0	0
**Category 5**									
Cryptosporidiosis	11	109	8	92	12	18	6	10	17
Giardiasis	137	113	103	94	86	86	53	73	70
**Total **	240	334	186	277	179	179	156	165	155
**Companion animal-borne zoonoses reported by veterinarians ** *									
Echinococcosis in dogs	ND	ND	ND	0	5	2	1	1	2
**Total **	ND	ND	ND	0	5	2	1	1	2

## 4. Unregulated and Emerging Zoonoses

The companion animal-borne zoonoses that must be reported and monitored in accordance with the Rabies Prevention Law and the Infectious Diseases Control Law have been widely observed and thoroughly investigated, both pathogenetically and epidemiologically, in past studies. While these diseases appear to be systematically controlled, emerging companion animal-borne zoonoses that are not required to be reported and monitored by either law and that have been less well understood have been reported in Japan. Drawing on the few data available, the outbreak status of these emerging zoonoses in Japan is discussed below.

### 4.1. Toxocariasis

Toxocariasis (visceral or ocular larval migrans) is caused by *Toxocara* species that parasitize in animals, such as *Toxocara canis* and *Toxocara cati*, which are parasitized in dogs and cats, respectively, and viewed with suspicion. Although epidemiological data regarding outbreaks of this disease between 1980 and 1990 have been reported, as described below, few data for 2000 or later have been reported. As such, the current status of toxocariasis in Japan is unclear.

#### 4.1.1. Epidemiology in Humans

Although humans are accidental hosts rather than the targets of *Toxocara* species, infection can result in visceral larva migrans and ocular larva migrans in humans. Although, as shown in Table 5, the number of humans infected with toxocariasis increased gradually between 1965 and 1990 in Japan [27], the true number may be even higher due to the possibility of asymptomatic infection and undiagnosed cases.

**Table 5:** Human outbreaks of toxocariasis in Japan. A dash indicates that the data were unavailable. Numerical data show the number of patients.

Fiscal year	Males	Females	Unknown	Total	Ocular larval migrans
Toxocariasis caused by *T. canis*					
1965–1970	3	4	-	7	3
1971–1980	6	-	-	6	6
1981–1985	8	8	10	26	13
1986–1990	24	20	4	48	37
1991–2011	7	2	-	9	7
Total	48	34	14	96	62
Toxocariasis caused by *T. cati*					
1975–1980	1	4	-	5	-
1981–1985	1	-	-	1	-
1986–1990	11	4	-	15	3
Total	13	8	-	21	3

#### 4.1.2. Epidemiology in Dogs and Cats

The prevalence of *Toxocara* species in dogs and cats in Japan ranges from 2.1% (adult) to 98% (young) in dogs and from 8.6% (adult) to 81.3% (young) in cats [27]. The trend of higher prevalence in young animals has been found in other countries [27].

### 4.2. Salmonellosis

Although most cases of salmonellosis, which is caused by *Salmonella* spp., are due to transmission by ingestion of contaminated food, the disease may also be transmitted by companion animals. After the first case of transmission by a turtle to an infant was reported in 1975 [28], companion animal-borne salmonellosis was recognized as social problem in Japan.

#### 4.2.1. Epidemiology in Humans

Several recently reported cases of human salmonellosis provide a perspective on those most at risk of infection and the route of transmission. These cases include an infant who developed diarrhea after possible transmission from an iguana in 2004, two children who developed severe salmonellosis after possible transmission from a red-eared slider turtle in 2005, a child who developed *Salmonella* enteritis after possible transmission from a red-eared slider turtle in 2005, an infant who developed *S* *almonella* septicemia after possible transmission from an African spurred tortoise (*Geochelone sulcata*) in 2006, and nine children who became infected with *Salmonella* Poona after possible transmission from reptiles in 2009 [9]. Although these reports suggest a reptile-to-human route of salmonellosis transmission, they do not provide definitive evidence of transmission of *Salmonella* spp. from animals to humans. The Ministry of Health, Labor and Welfare submitted a notice calling for increased attention to reptile-associated salmonellosis in 2005, however, the incidence of human infection and related clinical variables, such as the rate of serious infection, have not been definitively investigated and reported.

#### 4.2.2. Epidemiology in Reptiles

As described in Section 2.2, many imported red-eared slider turtles have recently been sold as pets in Japan, leading to speculation that the risk of *Salmonella* spp. infection from imported turtles is currently considerably high. In fact, sporadic outbreaks of human salmonellosis possibly transmitted from reptiles have been reported since 1975. Recent studies have reported detection of a high rate of *Salmonella* spp., which causes human salmonellosis, in red-eared slider turtles. One study detected *Salmonella* spp. in 188 of 227 turtles (83%) purchased from 29 pet shops between 2006 and 2008 [12]. One 2008 study reported that *Salmonella* spp. had been isolated in 37 of 115 animals (32.2%) kept in the home, 80 of 100 animals (80.0%) sold in pet shops, and 51 of 91 animals (56.0%) immediately after import [29].

### 4.3. Capnocytophagosis

Capnocytophagosis is caused by *Capnocytophaga* spp., resident bacteria in the oral cavity of dogs and cats that can transmitted from animals to humans by bites and scratches. Of the 14 individuals infected with capnocytophagosis between 2002 and 2009 in Japan, all of whom were between 40 and 100 years of age and also suffered from chronic diseases, such as diabetes, cirrhosis of the liver, and/or autoimmune disorders, six ultimately died [30]. A very low incidence of infection and a higher infection rate among those suffering from chronic diseases have also been reported in The Netherlands [31]. Although the incidence of capnocytophagosis is very low, high-risk groups, particularly those with chronic diseases, should be aware how capnocytophagosis is caused and how to prevent it. Physicians also should be aware of prompt testing for those groups.

### 4.4. Corynebacterium ulcerans *Infection*

*C. ulcerans* is related to *C. diphtheria*, which causes diphtheria. Toxigenic *C. ulcerans*, which infrequently produces the diphtheria toxin, produces diphtheria-like symptoms in infected humans. In reaction to emerging concerns regarding the role of this bacterium in human disease and companion animals as a potential source of infection, the Ministry of Health, Labor and Welfare submitted notices of a request for information to medical institutions in 2002 and 2009 [32].

#### 4.4.1. Epidemiology in Humans

The epidemiology of human *C. ulcerans* infection is not well understood [33]. In Japan, eight cases of *C. ulcerans* infection in humans were officially reported between 2001 and 2010. As can be observed in Table 6, which presents detailed information on these eight cases, most patients were 50 to 60 years of age and almost all kept companion animals [32].

**Table 6:** Cases of human *C. ulcerans* infection in Japan.

Date reported	Sex/age of case	Main symptom	Relationship with companion animals and related data	Toxinogenicity
Feb. 2001	Female/52	Breathing difficulty	Kept 20 cats, became symptomatic after death of one cat.	+
Oct. 2002	Male/54	Throat pain	Unknown, living in the same area as the above case	+
Sept. 2005	Male/58	Swelling of left parotid gland	After pet dog died, became symptomatic	+
Oct. 2005	Male/51	Many cavitary lesions in lung	Kept 12 cats	+
July 2006	Female/57	Throat pain	Kept companion birds	+
Jan. 2009	Female/57	Throat pain	Kept dogs and cats *C. ulcerans* isolated from cat	+
2010	Male/50s	Enlarged left axillary lymph nodes(no respiratory manifestations)	*C. ulcerans* isolated from cat	+
2010	Female/51	Throat pain	*C. ulcerans* isolated from cat	+

In Japan, vaccination with the diphtheria vaccine is required four times between the ages of three to 90 months (given as a combination of diphtheria, pertussis, and tetanus vaccines) and one time between the ages of 11 to 12 years (given as a combination of diphtheria and tetanus vaccines). This means of vaccination is considered effective for preventing diphtheria, but less for *C. ulcerans* infection because the infection in humans has been reported, as described above. Further research is required to clarify the impact of diphtheria vaccine on prevention of *C. ulcerans* infection.

#### 4.4.2. Epidemiology in Animals

Although the prevalence of toxigenic *C. ulcerans* in livestock and wild animals has been thoroughly investigated and reported, its prevalence in companion animals has become the true concern after reports of its isolation in cats [34] and dogs [35] in 2005. In Japan, toxigenic *C. ulcerans* was isolated from a dog for the first time in 2008 [36], and now requires further epidemiological investigation.

## 5. Prospects for the Control of Companion Animal-Borne Zoonoses in Japan

The risk of a zoonosis outbreak in Japan has increased due to recent social and global trends, such as the increased international movement of people and animals, ecological and environmental changes and degradation [37,38], and increased companion animal ownership [1]. When the increased risk of outbreak first began to be seriously investigated, the focus was transmission of zoonoses from livestock and wildlife. While certainly of importance, this focus does not encompass all means of zoonosis transmission, particularly as many people in developed countries live far from livestock and nature and have much contact with companion animals [1]. Recognizing this fact, companion animal-borne zoonoses have come under more scrutiny in developed countries.

Since the risk of outbreak of disease is influenced by human behavior, the prospects for disease control should be discussed at different levels of various institutions, including governmental, medical, veterinary and research institutions, as well as within the public and private spheres of daily life. The following sections discuss the role of public administration in future disease control in Japan.

### 5.1. Evaluating Current Regulation Systems

In common with other developed countries, the Japanese population faces increased risk of transmission of companion animal-borne zoonoses by increased contact with a greater number and variety of companion animals due to recent lifestyle and social trends. Japan also faces the challenges of a low dog registration rate and a high dog-bite injury rate, with approximately 5,000 dog bite injuries reported each year. In response, the Japanese government has implemented countermeasures for the domestic and international control of stipulated zoonoses in humans and companion animals under the Rabies Prevention Law and the Infectious Diseases Control Law. The government should now implement measures for assessing these regulation systems to determine whether they have met reasonable goals. Setting of these goals should be based on analysis of past companion animal-borne zoonosis outbreaks; cost-benefit considerations; and consensus formation among governmental, medical, veterinary, and research institutions. Although rabies has been well controlled, as evidenced by the fact no case of human and animal rabies has been reported over the past 50 years, it is not currently possible to determine whether the seven stipulated diseases—brucellosis, echinococcosis, leptospirosis, psittacosis, Q fever, cryptosporidiosis, and giardiasis—have been adequately controlled because the true number of zoonosis outbreaks caused by companion animals ownership is unclear (Section 3.3). Active surveillance of the seven diseases should be conducted on a national level to collect the data necessary to make this determination and identify trends in zoonosis transmission.

From December 2004 to March 2005, and January to February 2006, the specific program to evaluate the efficacy of countermeasures under the Infectious Diseases Control Law was conducted across the office and ministries in Japan, however this program focused on the countermeasures against severe infectious diseases of “Category 1” and “Category 2” stipulated by the law, not companion animal-borne zoonoses of “Category 4” and “Category 5” [4]. Given also that a framework for the regular evaluation of the infectious disease control system has not yet been established in Japan [4], Japanese ministries and other stakeholders should develop an adequate evaluation system to determine an appropriateness of the control system and improve it.

### 5.2. Policy Planning for Emerging Disease Control

It is also necessary for the Japanese government to determine which emerging companion animal-borne zoonoses diseases should be targeted by the Infectious Diseases Control Law and develop measures and policies for their control. Risk assessment of each disease should encompass consideration of such factors as the extent of its pathogenicity in humans, the possibility of medical treatment for the human form of infection, the epidemic situation in Japan and other countries, and the number of imported animals that might be vectors or reservoirs. Given that insufficient epidemiological data have been collected regarding emerging zoonoses, including toxocariasis, reptile-associated salmonellosis, capnocytophagosis, and *C. ulcerans* infection, as discussed in Section 4, active national surveillance of the diseases should be established as a high priority. Collecting detailed epidemiological data regarding these diseases in Japan is essential in policy planning for disease control in Japan.

Once the target zoonoses are chosen, countermeasures against the diseases should be conducted more actively under the supervision of the Japanese government. Using the laws established in other countries as models for new regulations may prove valuable. For example, since 1975, the Food and Drug Administration has enforced a regulation in the U.S. that bans the sale of turtles with a carapace length of less than four inches to prevent turtle-associated salmonellosis [39]. Introduction of such a regulation into the zoonosis control system in Japan may serve as a valuable disease control measure.

## 6. Conclusions

While ownership of animals provides companionship and its associated benefits for humans, it also poses the risk of transmission of certain diseases. To tilt the balance between the risks and benefits of pet ownership toward the former, it is necessary to obtain a correct understanding of the true status of zoonosis outbreaks, particularly of emerging diseases. As discussed in this report, Japan’s approach to the control of companion animal-borne zoonoses contributes to the creation of a social system that keeps humans and animals healthy and vigorous. As such, it provides a template for the development of further countermeasures against animal-borne diseases throughout the world. To develop these countermeasures, researchers must endeavor to collect the epidemiological data necessary for the development of regulations for preventing and controlling zoonosis outbreaks within both the public and private spheres at all levels, including governmental, medical, veterinary, and educational. Collection of such epidemiological data will also contribute to disease control and prevention at the international level.

**Conflict of Interest**

The authors declare no conflict of interests.
